# Clinical outcomes of transforaminal versus interlaminar epidural injection among patients with lumbosacral disc herniation

**DOI:** 10.6026/973206300213170

**Published:** 2025-09-30

**Authors:** Jaideep Singh, Gautam Chandra Koshi, Sakshi Shukla, Kaushal R.P.

**Affiliations:** 1 Department of Anesthesiology, Gandhi Medical College, Bhopal, Madhya Pradesh, India

**Keywords:** Lumbosacral disc herniation, transforaminal epidural steroid injection, interlaminar epidural steroid injection, pain management

## Abstract

A clinical outcome of the Transforaminal Epidural Steroid Injection (TFESI) and Interlaminar Epidural Steroid Injection (ILESI) procedures in patients with lumbosacral disc herniation is of interest. Hence, a total of 60 patients were randomly allocated to be given TFESI (n=30) or ILESI (n=30). The two groups were both administered 8 mg dexamethasone and 0.5 ml of preservative-free lignocaine filled to the 1ml level. Visual Analog Scale (VAS) and the Numeric Rating Scale (NRS) are to be used to measure pain at the pre-intervention, 15 days of intervention, as well as 3 months post-intervention. Thus, we show that TFESI was effective with enhanced pain relief, especially at the three-month follow-up.

## Background:

The problem of lumbosacral disc herniation is a widespread disease of the spine that is the cause of extreme pain, disability and impaired quality of life for millions of people in the world. It is an event that takes place when the inner nucleus pulposus of an intervertebral disc bulges through the relatively weakened or torn outer annulus fibrosus; this is likely to compress any surrounding nerve roots [1]. The evident ensuing inflammation and mechanical stress on the neural structures usually translate to the radiating pain, numbness and weakness in the lower limbs, not forgetting that low back pain is debilitating [2]. Treatment of lumbosacral disc herniation includes all types of therapeutic functions, including physical therapy, oral medications and surgery at the surgical level 3]. Epidural steroid injections have become one of the most popular treatment methods among other modalities because of their non-invasive nature and being a less damaging alternative to those patients who have not responded well to more conservative therapies but are not yet qualified to have surgery [4]. The purpose of these injections is to administer powerful anti-inflammatory corticosteroid agents and local anesthesia into the area and decrease inflammation, pain and possibly improve the functional outcome [5]. There has arisen two main methods of epidural injection in the lumbosacral area namely the transforaminal and interlaminar methods. They are all different in their anatomical considerations, the possible benefits and the risks involved. Transforaminal is the method of injection in which the medication is injected inside the neural foramen, through which the spinal nerve leaves the spinal canal [6]. The method is believed to permit more accurate targeting of the delivery of the therapeutic agents to the therapeutic space often affected by the pathology in disc herniations in the ventral epidural space [7]. On the other hand, the interlaminar approach requires one to insert the needle into the region between the laminae of two vertebrae and gain access to the dorsal epidural space, where the medication may spread to cover a larger area.

The apparent controversy about the superiority of efficacy and safety of these two approaches has led to a multitude of clinical trials and meta-analyses [8, 9]. Advocate of the transforaminal method claim that it allows a more localized delivery of medication to the ventral epidural space and the regions of the distressed nerve root, with a possible improvement of pain relief and functional outcome. Also, with the fluoroscopic guidance of transforaminal injections, there is the possibility that the accuracy of needle positioning can be enhanced and unintended intravascular injection can be avoided [10]. Conversely, proponents of the interlaminar approach outline its potential benefits, which have included a reduced chance of some of the complications typical to the method, i.e., intravascular injection and spinal cord injury [11]. The interlaminar technique could also be favored to treat patients with difficult anatomy or patients with multilevel pathology because more medication can be dispersed throughout the epidural space during this technique [12]. Although evidence behind this question has been small in number, it remains that no optimal way of carrying it out when it comes to epidural steroid injections on patients with lumbosacral disc herniation has been reached [13].

This continuous confusion underlines the necessity of carefully conducted comparative experiments which have to directly evaluate the clinical effect of the transforaminal injection intralaminar injection in the said unique group of patients. The current research will fill this research gap or lack of knowledge by doing an in-depth comparison of the outcomes of transforaminal epidural injection and interlaminar epidural injection response rates in patients with complaints of lumbosacral disc herniation. In estimating the significance of the main parameters used in this study, which include the effect of the intervention on pain and improvement of its function, patient satisfaction and the rate of complication occurrence, this study aims to contribute valid insights that may play in regulating clinical decisions and support the best in patient care [14]. The importance of the study lies in the possibility of its use in the development of evidence-based practice in the treatment of lumbosacral disc herniation. Precisely, given the fact that healthcare systems across the world are dealing with the augmented socioeconomic burden of spinal disorders [15], it is especially important to determine the safest and most efficient interventional methods. Furthermore, during the era of personalized medicine, the knowledge of the comparative advantages of transforaminal and interlaminar procedures can help to provide more patient-specific options that consider both patient-specific and patient-preferred approaches [16]. Other significant clinical questions asked as part of this research also have additional implications beyond the immediate comparison of the techniques of injection. As an example, it could illuminate the preference time of epidural injections during the journey of disc herniation, how long the effects of the treatment continue and whether it could have any effect on the necessity of such surgical intervention [17]. Another contribution of the study is related to the fact that improperly documented adverse events and complications related to each treatment method provide the basis to continue improving safety standards and risk reduction measures during interventional pain management [18]. Moreover, such an inquiry can make a difference in the healthcare policy and resource distribution. Given the rise in the focus on cost-effectiveness and value-based care in healthcare systems, it is of great concern to know which of the various interventional methods is most effective [19]. The results of this research can contribute to the establishment of recommendations on how epidural injection can be used appropriately, which can result in a more cost-effective use of medical care resources and patient survival [20]. Therefore, it is of interest to evaluate the clinical outcomes of transforaminal versus interlaminar epidural injection in patients with lumbosacral disc herniation.

## Methodology:

## Study design:

This prospective, randomized, double-blind, controlled trial was designed to compare the clinical outcomes of TFESI versus ILESI in patients with lumbosacral disc herniation.

## Participants:

Sixty patients (aged 25-65 years) with radiologically confirmed lumbosacral disc herniation and corresponding radicular pain were recruited from the pain management clinic of our institution. Inclusion criteria were: (1) radicular pain lasting for at least 6 weeks, (2) failure of conservative management and (3) a score of ≥4 on both the Visual Analog Scale (VAS) and Numeric Rating Scale (NRS) for pain. Exclusion criteria included previous lumbar surgery, cauda equina syndrome, progressive neurological deficits and contraindications to epidural steroid injections.

## Randomization and blinding:

Patients were randomly assigned to either the TFESI group (n=30) or the ILESI group (n=30) using a computer-generated randomization sequence. Both patients and outcome assessors were blinded to the treatment allocation. The physician performing the procedure was not blinded due to the nature of the interventions.

## Intervention:

All procedures were performed under fluoroscopic guidance by experienced pain specialists. The TFESI group received 8mg of dexamethasone and 0.5 ml of preservative-free lignocaine diluted to 1mlvia the transforaminal approach, while the ILESI group received the same medication mixture via the interlaminar approach. A single injection was administered for each patient at the level corresponding to the disc herniation.

## Outcome measures:

The primary outcome measures were:

[1] Visual Analog Scale (VAS) for pain (0-10 cm)

[2] Numeric Rating Scale (NRS) for pain (0-10)

Assessments were conducted at pre-intervention, 15 days and 3 months post-intervention. Demographic data, including age, gender and weight of participants, were collected at baseline.

## Data collection:

VAS and NRS scores were recorded at each assessment point. Patients were also monitored for any adverse events throughout the study period. To minimize loss to follow-up, reminder calls were made before each scheduled visit.

## Statistical analysis:

Sample size was calculated to detect a clinically significant difference of 2 points on both VAS and NRS between groups, with 80% power and a 5% significance level. Demographic characteristics were compared using t-tests for continuous variables and chi-square tests for categorical variables. Pain scores (VAS and NRS) were analyzed using independent t-tests to compare between groups at each time point. Paired t-tests were used to assess changes in pain scores within each group from baseline to follow-up time points. A p-value < 0.05 was considered statistically significant.

## Ethical considerations:

The study protocol was approved by the institutional ethics committee. Written informed consent was obtained from all participants before enrolment in the study. The trial was registered with the appropriate clinical trials registry.

## Data management:

All data were entered into a secure, password-protected database. Double data entry was performed to ensure accuracy. Only de-identified data were used for analysis to maintain patient confidentiality. Table 1 (see PDF) shows that there are no statistically significant differences in age, gender distribution, or weight between the TFESI and ILESI groups. This indicates that the groups are well-matched at baseline, which is important for comparing treatment outcomes. Table 2 (see PDF) demonstrates that while there were no significant differences in baseline pain scores, the TFESI group showed significantly lower pain scores at both 15 days and 3 months post-intervention for both VAS and NRS measures. The difference is particularly pronounced at the 3-month follow-up. Table 3 (see PDF) shows the pain reduction from pre-intervention to 3 months. While the VAS reduction was not significantly different between the two groups, the NRS reduction was significantly greater in the TFESI group. This suggests that TFESI may be more effective in reducing pain when measured using the NRS scale. The statistical analysis reveals that both TFESI and ILESI are effective in reducing pain associated with lumbosacral disc herniation. However, the TFESI group demonstrated significantly better pain reduction at both 15 days and 3 months post-intervention, particularly when measured using the NRS. The lack of significant difference in VAS reduction from pre-intervention to 3 months, contrasted with the significant difference in NRS reduction, suggests that the NRS may be more sensitive to detecting differences in pain reduction between these two techniques.

## Results and Discussion:

This article was based on a comparison of clinical activity of transforaminal epidural steroid injections (TFESI) and interlaminar epidural steroid injections (ILESI) among patients who had lumbosacral disc herniation. These results prove that both methods are useful to decrease the number of pain, but TFESI proved to be more effective, especially 3 months after the procedure. A similar protocol by Chen *et al.* also compared transforaminal and interlaminar approaches and outlined key procedural considerations supporting their clinical equivalence and safety profile [21]. The considerable level of pain relief in the two groups is consistent with other studies that have indicated an epidural steroid injection to be a viable treatment option in the reduction of radicular pain caused by disc herniation [4, 5]. Nonetheless, conclusions of Chang-Chien *et al.* regarding better results in the transforaminal method [9] are supported by the preferable effects of TFESI, particularly in the latest NRS results. This better performance of TFESI can be explained by a more specific delivery of medication to the ventral epidural space. This accurate application will automate a higher concentration of the steroid at the injury point, where the effects may cause increased anti-inflammatory responses along with improved pain relief. Interestingly, both VAS and NRS were found to be significant among the groups at 15 days and 3 months, but it seems NRS was more sensitive to identify the differences between the groups. Such a finding begs the question concerning the comparative sensitivity of these instruments for measuring pain when it comes to epidural injections, which is a discussion that has to be further researched. Both methods of our study had a favourable safety profile and no major complications occurred. This agrees with the conclusion of Rathmell *et al.* (2015), wherein the research group stressed how epidural steroid injections are generally safe as long as they are administered by skilled doctors following the established necessary precautions [18]. Although TFESI had better short-term results, it is interesting to mention that both methods were found to have significant pain relief on a clinical scale. This concurs with the overall positive use of epidural steroids in the treatment of radicular pain as indicated in the systematic review provided by Lee *et al.* [8]. There are a number of limitations to our study. The 3-month follow-up could possibly lack long-term results and this is one aspect that should be considered by a future study. We also failed to assess the functional outcomes and quality of life indicators that could give a better picture of the clinical value of such interventions. There are also clinical implications of the findings of this study. TFESI and ILESI are possible alternatives in treating radicular pain in cases of lumbosacral disc herniation, but the high level of pain relief by TFESI justifies that it could be the way forward, especially in severe cases or even in patients who have been resistant to alternative conservative measures of treatment. In the future, trials ought to be directed to longer-term results, the feasibility of repeated injections and the cost-effectiveness of such methods. Furthermore, clinical decision-making can be improved even more by conducting research studies, which examine the optimal timing of epidural surgery in the context of disc herniation, as proposed by Bicket *et al.* [17].

## Conclusion:

Both TFESI and ILESI yield success in lessening pain among patients encountering lumbosacral disc herniation. Nevertheless, TFESI had better pain relief, especially in the 3-month follow-up. Thus, we show the application of TFESI as an option of choice in the treatment of radicular pain caused by a herniated lumbosacral disc in situations where incorporation of a medicine through direct delivery becomes essential.

## References

[1] Fardon DF (2014). Spine J..

[2] Ropper AH, Zafonte RD (2015). N Engl J Med..

[3] Kreiner DS (2014). Spine J..

[4] Manchikanti L (2014). Pain Physician..

[5] Cohen SP (2015). BMJ..

[6] McLain RF (2005). Spine J..

[7] Ghahreman A (2010). Pain Med..

[8] Lee JH (2018). Pain Physician..

[9] Chang-Chien GC (2014). Pain Physician..

[10] Manchikanti L (2010). Pain Physician..

[11] Gharibo CG (2011). Pain Physician..

[12] Rados I (2011). Pain Med..

[13] Kennedy DJ (2014). Pain Med..

[14] Manchikanti L (2013). Pain Physician..

[15] Hoy D (2014). Ann Rheum Dis..

[16] Park CH (2010). Pain Med..

[17] Bicket MC (2013). Anesthesiology..

[18] Rathmell JP (2015). Anesthesiology..

[19] Manchikanti L (2014). Pain Physician..

[20] Chou R (2015). Ann Intern Med..

[21] Chen W (2020). Medicine (Baltimore)..

